# Investigation of *Leishmania infantum* Infection and Feeding Preferences of *Lutzomyia longipalpis* During Deltamethrin (4%) Dog Collar Intervention

**DOI:** 10.3390/pathogens14070671

**Published:** 2025-07-08

**Authors:** Gabriel F. F. Rodrigues, Keuryn A. M. Luz-Requena, Bruno S. Mathias, Tania M. T. Suto, Rosemari Suto, Luciana T. R. Rocha, Osias Rangel, Katia D. S. Bresciani, Susy M. P. Sampaio, Lilian A. C. Rodas, Karin Kirchgatter

**Affiliations:** 1 Programa de Pós-Graduação em Doenças Infecciosas e Saúde Global, Faculdade de Medicina, Universidade de São Paulo/USP, São Paulo 05403-000, SP, Brazil; gfferrari@usp.br (G.F.F.R.);; 2 Programa de Pós-Graduação em Ciência Animal, Faculdade de Medicina Veterinária, Universidade Estadual Paulista/UNESP, Araçatuba 16050-680, SP, Brazil; kaml.requena@unesp.br; 3 Coordenadoria de Controle de Doenças, Secretaria da Saúde do Estado de São Paulo, São Paulo 01246-000, SP, Brazil; 4 Prefeitura Municipal de Mariápolis, Mariápolis 17810-000, SP, Brazil; 5 Seção de Vetores-Campinas, Instituto Pasteur, Campinas 13041-025, SP, Brazil; 6 Departamento de Produção e Saúde Animal, Faculdade de Medicina Veterinária, Universidade Estadual Paulista/UNESP, Araçatuba 16050-680, SP, Brazil; katia.bresciani@unesp.br; 7 Seção de Vetores-Presidente Prudente, Instituto Pasteur, Presidente Prudente 19013-050, SP, Brazil; 8 Seção de Vetores-Araçatuba, Instituto Pasteur, Araçatuba 16015-160, SP, Brazil; 9 Laboratório de Bioquímica e Biologia Molecular, Instituto Pasteur, São Paulo 01027-000, SP, Brazil

**Keywords:** vectors, blood source, *Leishmania*, sand flies, leishmaniasis

## Abstract

Leishmaniasis is a zoonotic disease caused by protozoa of the genus *Leishmania*, transmitted by phlebotomine sand flies. Understanding the feeding behavior and infection rates of these vectors is crucial for disease surveillance and control. We aimed to investigate the natural infection rate of *Leishmania* spp. in phlebotomines and analyze their blood-feeding patterns in one of the priority areas of the state of São Paulo for the implementation of insecticide-impregnated dog collars. Sand flies were collected from urban and peri-urban areas between 2022 and 2024 using CDC light traps, manual aspiration, and Shannon traps. PCR was used to detect *Leishmania* DNA (SSU rDNA gene), and blood meal sources (COI gene). A total of 414 sand flies were collected, with 222 engorged females analyzed for blood meals and 192 specimens tested for *Leishmania* spp. infection. The predominant blood source was humans (67%), followed by chickens (64.1%), and dogs (18.9%), considering that 45.1% of the samples presented mixed blood meals. *Leishmania infantum* was found in 1% of the samples. These findings highlight the feeding plasticity of sand flies and their potential role in disease transmission, reinforcing the need for continuous epidemiological surveillance and vector control strategies, particularly the implementation of insecticide-impregnated dog collars.

## 1. Introduction

Leishmaniasis is an enzootic and zoonotic disease caused by parasitic protozoa of the genus *Leishmania* (Trypanosomatida: Trypanosomatidae) and transmitted through the bite of phlebotomine sand flies (Diptera: Psychodidae). The parasite has a life cycle that alternates between mammalian hosts, including humans, and sand fly vectors, specifically those of the *Phlebotomus* genus in the Old World and the *Lutzomyia* genus in the New World (rev. in [1]).

Leishmaniasis is a chronic and often fatal zoonotic disease, recognized as a global public health concern. The World Health Organization (WHO) classifies it as one of the neglected tropical diseases [2]. In 2018, tegumentary leishmaniasis (TL) was considered endemic in 92 countries, while human visceral leishmaniasis (HVL) was reported in 83 countries [3]. Of the 11 countries that report 90% of TL cases globally, 3 are in the Americas: Brazil, Colombia, and Peru. For HVL, 4 countries account for 60% of cases globally: Brazil, Ethiopia, Kenya, and Sudan [4].

In Brazil, dogs are the most important reservoirs of the parasite and serve as the blood-feeding source for sand flies in urban areas. Similar to humans, infected dogs can develop clinical disease, leading to canine visceral leishmaniasis (CVL), which can be fatal. The occurrence of human cases is closely associated with the presence of infected dogs [5,6,7,8].

In the state of São Paulo, the municipality of Mariápolis was designated by the Brazilian Ministry of Health as a priority area for the implementation of insecticide-impregnated dog collars (deltamethrin 4%), as established by the National Program for Surveillance and Control of Visceral Leishmaniasis. This designation was based on the annual average number of HVL cases reported between 2019 and 2021 in the state [6,9,10].

In Mariápolis, the presence of the vector *Lutzomyia longipalpis* was first identified in 2005. Two years later, seropositive dogs for CVL were diagnosed. However, the first human case was recorded in 2009, resulting in death within the same year. Since then, between 2018 and 2019, one case was reported each year, with no fatalities and an incidence rate of 24.4%. In 2020, three additional human cases were diagnosed, raising the incidence rate to 73.2%, though no deaths were reported [11]. Even without fatalities in recent years, the municipality is classified as high risk and designated as a priority area due to its average incidence rate, making it eligible for inclusion in the canine collar program.

Epidemiological and entomological surveys have always been essential for understanding the endemic dynamics of leishmaniasis and determining the relationship between vector species and reservoirs involved in wild and urban transmission cycles of *Leishmania* spp. Therefore, the blood-feeding patterns of phlebotomine and their infection status is crucial for identifying potential mammalian reservoirs and vector feeding preferences [12].

In this study, our objective was to investigate the rate of natural infection by *Leishmania* spp. in phlebotomine sand flies in the municipality of Mariápolis, São Paulo, Brazil, using molecular diagnosis targeting SSU rDNA. Additionally, we analyzed the blood-feeding patterns of phlebotomine sand flies. Through this analysis, we sought to address key questions related to the transmission dynamics of VL, emphasizing the critical role of epidemiological surveillance in controlling the spread of the disease.

## 2. Materials and Methods

### 2.1. Study Area

The study was carried out in the urban and peri-urban area of the municipality of Mariápolis (21°47′54″ S; 51°10′45″ W). The municipality is located in the state of São Paulo, in the Southeast region of Brazil. Covering an area of 186.098 km^2^ and situated at an elevation of 410 m, the town is part of the Marília Administrative Region.

The collaring of dogs in Mariápolis began in 2022, with an expected end date scheduled for 2026. The application of the collars was carried out in semiannual cycles, with the first cycle starting in August 2022, the second in February 2023, the third in August 2023, the fourth in February 2024, and the fifth cycle in July 2024. In total, 2529 dogs were collared, with an average of 509 dogs per cycle.

**Figure 1:**
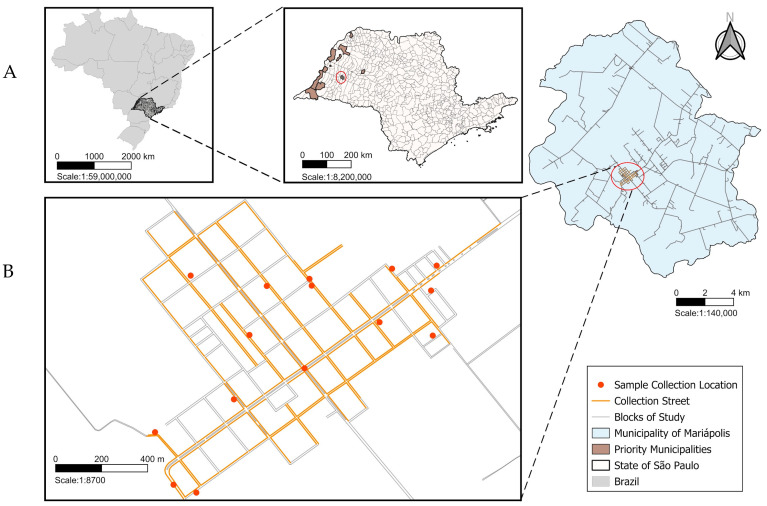
(**A**) Location of the state of São Paulo on the map of Brazil and location of the municipalities designated by the Brazilian Ministry of Health as priority areas for the implementation of insecticide-impregnated dog collars (Deltamethrin 4%), with highlight for Mariápolis (red circle). (**B**) Blocks of the municipality of Mariápolis where the sand flies were collected (red dots).

Houses in the urban area were selected for sand fly collection based on conditions favorable for the development of these insects. These included residences with extensive peridomestic areas, abundant vegetation (particularly dense shrubs or large, leafy trees, and accumulation of organic matter on the ground, such as leaves, fallen fruit, and animal feces), and the mandatory presence of dogs. Additionally, the presence of other animals, such as chickens and ducks, which could serve as potential blood-feeding sources for sand flies, was also considered. Figure 1 shows the distribution of the collection sites in the municipality and its location in the state of São Paulo, near other municipalities designated by the Brazilian Ministry of Health as priority areas for the implementation of insecticide-impregnated dog collars (deltamethrin 4%).

### 2.2. Entomological Capture and Sand Fly Identification

**Figure 2:**
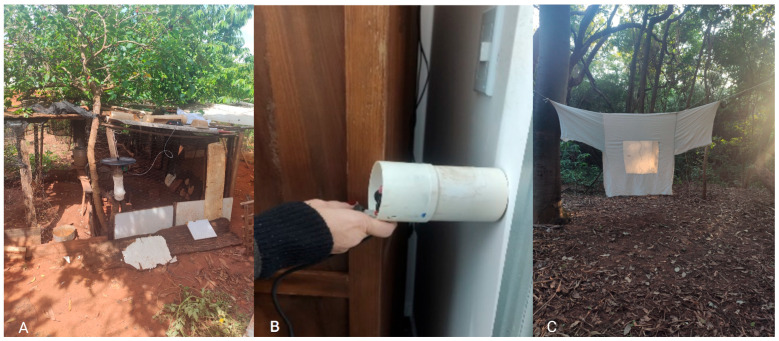
Capture methodologies: (**A**) CDC light traps; (**B**) manual capture using electric aspirators; and (**C**) Shannon tent trap.

The sand flies were collected between November 2022 and May 2024, during the evening twilight period. The collection techniques included CDC light traps [13], manual capture using electric aspirators [14], and Shannon traps [15] (Figure 2A–C), the latter being exclusively used in peri-urban areas. All sand fly collections occurred during the implementation of dog collaring cycles.

From the data collection period, monthly sampling was carried out in both intradomiciliary and peridomiciliary environments for three consecutive nights. CDC light traps were used for 12 h, starting at dusk, while manual capture was conducted for 20 min per house, also beginning at dusk and continuing until 10:00 pm. Taxonomic identification of sand flies was performed according to Galati [16]. The insect was placed in 1.5 mL micro tubes containing 100% ethanol and stored at −20 °C until DNA extraction, at the Biochemistry and Molecular Biology Laboratory of the Pasteur Institute.

### 2.3. DNA Extraction

The extraction of genomic DNA was performed by macerating each sand fly individually using the FastPrep-96 equipment (MP Biomedicals, Solon, OH, USA) in tubes containing 1.4 mm ceramic beads (Qiagen, Hilden, Germany) along with a 6.35 mm zirconium oxide bead (MP Biomedicals) in lysis buffer Master Mix [200 µL of nuclear lysis solution, 50 µL of 0.5 M EDTA (ethylenediaminetetraacetic acid) pH 8.0, and 5 µL of RNase A solution] for 4 min at 1800 rpm, followed by centrifugation for 5 min at 14,000 rpm at room temperature. Subsequently, 20 µL of proteinase K (20 mg/mL) was added and incubated for 16 h at 55 °C. The extraction continued using the commercial Wizard SV 96 Genomic DNA Purification System Kit (Promega, Madison, WI, USA). Lysates were transferred to the Binding Plate and washed according to the manufacturer’s instructions, using a vacuum pump at a pressure of 15–20 inches Hg. The DNA was eluted in 100 µL of nuclease-free water and stored at −20 °C until analysis.

### 2.4. PCR for Detection of Leishmania spp.

The extracted DNA was subsequently processed using a nested PCR protocol targeting SSU rDNA to detect and identify the parasite [17]. Each reaction had a final volume of 25 µL, including 0.5 units of Platinum™ *Taq* DNA Polymerase (Invitrogen by Thermo Fisher Scientific, Carlsbad, CA, USA), 0.2 mM of each dNTP, 0.2 µM of each primer, 2 mM MgCl_2_, 1X PCR buffer, and 2 µL of genomic DNA. The initial PCR utilized primers S4 (5′–GAT CCA GCT GCA GGT TCA CC–3′) and S12 (5′–GGT TGA TTC CGT CAA CGG AC–3′), as described by Uliana et al. [18]. The cycling protocol started with denaturation at 94 °C for 3 min, followed by 40 cycles of 94 °C for 1 min, 55 °C for 1 min, and 72 °C for 1 min, with a final extension at 72 °C for 7 min, producing 520 bp fragments. The product of this first PCR, amplified with primers S4 and S12, was then used as a template in a nested PCR with primers S17 (5′–CCA AGC TGC CCA GTA GAA T–3′) and S18 (5′–TCG GGC GGA TAA AAC CC–3′), specific to the *Leishmania* genus. This nested reaction was conducted under the same conditions, using the first PCR product diluted 1:10,000 in ultrapure water. The cycling for this nested PCR began with denaturation at 94°C for 4 min, followed by 35 cycles of 94 °C for 1 min, 60 °C for 1 min, and 72 °C for 30 s, with a final extension at 72 °C for 7 min, generating fragments of 490 bp. The amplified products were examined on a 1% agarose gel, stained with GelRed® (Biotium, Fremont, CA, USA).

In all the PCR amplifications, we took standard precautions to prevent cross-contamination of samples and included negative controls (ultrapure water without DNA) to check for possible contamination and false positives.

### 2.5. Sequencing

The nested PCR fragments obtained using the S17–S18 primers were purified with the EXOSAP-IT kit (Thermo Fisher Scientific Inc., Waltham, MA, USA) and sequenced using the BigDye^®^ Terminator v3.1 Cycle Sequencing Kit on an ABI PRISM^®^ 3500 Genetic Analyzer (Applied Biosystems, Waltham, MA, USA). Sequencing was performed in a multi-user facility at the Institute of Tropical Medicine (IMT), University of São Paulo (USP), using the same nested PCR primers. The target region contained single-nucleotide polymorphism (SNP) that enabled the differentiation of *Leishmania amazonensis*, *Leishmania infantum*, and *Leishmania guyanensis* [17]. The obtained sequences were aligned with GenBank sequences using the BLAST (Basic Local Alignment Search Tool) version 2.16.0.

### 2.6. Analysis of Sand Fly Blood-Feeding Sources

To identify human DNA as a blood meal source, oligonucleotides described by Parodi et al. [19] were used. For humans, primers F (5′ TTC GGC GCA TGA GCT GGA GTC C 3‘) and R (5′ TAT GCG GGG AAA CGC CAT ATC G 3′) were applied to amplify a 228 bp fragment of the cytochrome c oxidase I (COI) gene. For dogs, primers F (5′ GAA CTA GGT CAG CCC GGT ACT T 3′) and R (5′ CGG AGC ACC AAT TAT TAA CGG C 3′) amplified a 153 bp COI gene fragment, while, for chickens, primers F (5′ GGG ACA CCC TCC CCC TTA ATG ACA 3′) and R (5′ GGA GGG CTG GAA GAA GGA GTG 3′) amplified a 266 bp fragment of the COI gene. PCR conditions followed Chang et al. [20] with modifications: 2 mM MgCl_2_, 1X Taq buffer, 0.12 mM dNTP mix, 0.25 µM of each primer, and 0.5 U of Platinum™ *Taq* DNA Polymerase (Invitrogen by Thermo Fisher Scientific, Carlsbad, CA, USA). The thermal cycling program included an initial denaturation at 95 °C for 5 min, followed by 35 cycles of 95 °C for 30 s, (65 °C for human PCR, 67 °C for dog PCR; 69 °C for chicken PCR) for 30 s, and 72 °C for 30 s, with a final extension at 72 °C for 10 min. Modifications to the protocol included adjustments to reagent concentrations and annealing temperature. Amplified products were analyzed on a 1.5% agarose gel stained with GelRed® (Biotium, Fremont, CA, USA).

To amplify host blood DNA from engorged sand flies that tested negative for human, chicken, and dog DNA, the primers L14841 (5′ AAA AAG CTT CCA TCC AAC ATC TCA GCA TGA TGA AA 3′ and H1514923 (5′ AAA CTG CAG CCC CTC AGA ATG ATA TTT GTC CTC A 3′) were used as described by Kocher et al. [21], to target a 300 bp fragment of the mitochondrial *cytb* gene. This versatile protocol enables the identification of a broad spectrum of animal species.

### 2.7. Statistical Analysis

A proportion test was conducted to assess the natural infection rate using a population sample, chi-square distribution, and 95% confidence interval with Yates’ continuity correction. Similarly, the same tests were performed to construct confidence intervals for the collection methods. Additionally, a chi-square test was applied to analyze probability distributions of frequencies from consolidated collection data. All statistical analyses were performed using the R software version 4.5.1 with the stats library (https://www.R-project.org/ accessed on 19 June 2025).

## 3. Results

**Figure 3:**
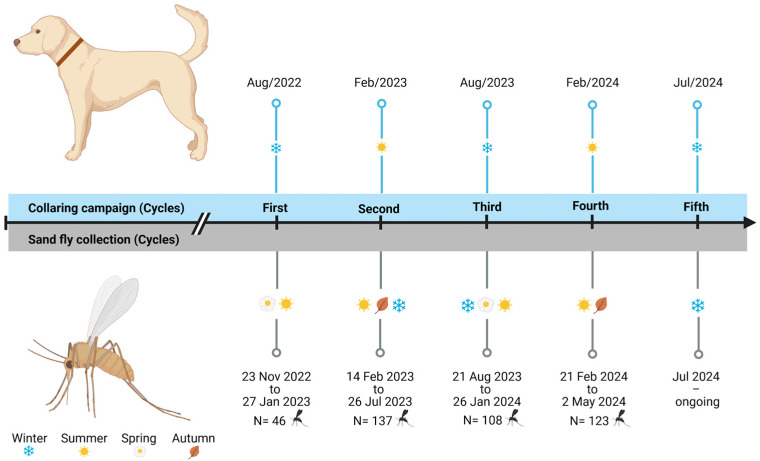
Dog collaring campaigns and sand fly collection timeline, Mariápolis, Brazil, 2022–2024. Created at https://BioRender.com (accessed on 19 June 2025).

A total of 414 female sand flies were collected between November 2022 and May 2024 (Figure 3, Table 1), with 222 females selected for blood meal source identification and 192 for *Leishmania* spp. infection analysis. All sand flies obtained were identified as *Lu. longipalpis*, except for the specimens collected with the Shannon trap: two *Nyssomyia whitmani* and two *Nyssomyia neivai*.

In relation to the capture methods used, a significant difference in collections using a manual electric aspirator was observed (Table 1). Almost all the engorged females (99.1%, 220/222) were obtained with manual captures, with only one specimen collected at CDC and one specimen collected at the Shannon trap. Similarly, regarding the total number of non-engorged females collected, the greatest production (82.3%, 158/192) was through manual aspiration. CDC electric traps collected 16.1% (31/192) of non-engorged females and Shannon traps 1.6% (3/192).

A total of 192 female sand fly specimens were individually analyzed for the presence of *Leishmania* spp. DNA. Eleven were collected in 2022, 128 in 2023 and 53 in 2024. This difference was reasonably proportional to the number of months of captures. In 2022, it was two months, 2023 the whole year, and 2024 four months. Among these, two tested positive corresponding to an infection rate of 1.0% (CI 95% = 0.18–4.1%) at the same location address and using the same capture method (electric aspirators) in different years, specifically in June 2023 and January 2024. The samples were sequenced and analyzed by BLASTn in the GenBank database, showing a 100% similarity with *Leishmania infantum* (OL616091, isolate MHOM/BR/PP75 [22]).

Among the 222 female sand flies collected with blood meals, the origin was successfully determined for 206 specimens using COI or *cytb* PCRs. Identification failed in 16 individuals, possibly due to the small volume of blood ingested by the insect, or alternative sources such as sap, or due to the degradation of the blood within the sand flies (Table 2).

Of the engorged females successfully analyzed, 54.8% (113/206) had a single blood meal source (Table 2). Among these, chicken was the most prevalent, with 50 (44.2%) sand flies feeding on them, followed by humans with 48 (42.5%), dogs with 8 (7.1%), and ducks with 7 (6.2%). However, 76 out of 206 sand flies (36.9%) had fed on two types of hosts, with humans and chickens being the most common meal feed at 81.6%, followed by dogs and humans at 14.5%, and dogs and chickens at 3.9% (Table 2). Additionally, 17 out of 206 sand flies (8.3%) had fed on three different hosts: dogs, humans, and chickens. However, looking at the presence of blood in all the sand flies with an identified food source as a whole, we can see that human blood was the most found (67%, 138/206), followed by chicken (64%, 132/206) and very little dog blood (19%, 39/206).

During the identification of the species, an engorged male sand fly was observed, prompting the application of the blood meal identification protocol, which confirmed chicken as the source of the blood meal.

## 4. Discussion

Entomological surveys play a crucial role in elucidating the transmission dynamics of leishmaniasis and identifying the relationships between vector species and potential reservoirs involved in the *Leishmania* spp. cycle. In this study, we investigated the blood-feeding patterns and infection status of phlebotomine sand flies in Mariápolis, a priority intervention area where insecticide-impregnated dog collars have been deployed. Between November 2022 and May 2024, a total of 414 sand flies were collected, with 222 females analyzed for blood meal source identification and 192 for *Leishmania* spp. infection. Two females tested positive for *Leishmania infantum*. Blood meal sources were successfully identified in 206 engorged females, revealing a predominance of human (67%) and chicken (64%) blood, with dog blood detected in 19% of cases. Mixed blood meals were observed in 45.2% of the specimens.

Previous studies have demonstrated that deltamethrin-impregnated dog collars can potentially reduce the proportion of *Leishmania*-infected sand flies in vector populations [23,24,25]. However, we observed an *L. infantum* infection rate of 1% in sand flies. This rate is comparable to those reported in other leishmaniasis-endemic municipalities in São Paulo State, such as Panorama, Valinhos, Andradina, and Ilha Solteira, where no canine collar programs were in place [26,27,28]. It is also noteworthy that, in other Brazilian states—including Bonito (MS), Janaúba (MG), Várzea Grande (MT), São Luís (MA), and Mossoró (RN)—infection rates in known *L. infantum* vectors are similarly low, typically ranging from 0.25% to 1.8% [29,30,31,32,33]. Although direct comparisons are appealing, they must be interpreted with caution, as the observed variations may reflect not only differences in the sensitivity and specificity of the detection methods used, but also the intrinsic spatial variation in natural infection rates. These differences can be influenced by a range of factors, including sampling strategies, host availability, local parasite prevalence, and broader environmental or ecological conditions.

A unique observation in our study was the presence of an engorged male sand fly, an unusual finding since blood-feeding is typically restricted to females for egg development. Although rare, this behavior has been reported in other families of the Diptera order [34,35], which demonstrates that, under certain conditions, male mosquitoes may consume blood. According to the authors, while this behavior is atypical, it cannot be entirely ruled out, as it may occur depending on environmental factors and the availability of food sources [35]. Additionally, a study conducted in the Southeastern region of Brazil reported variations in feeding behavior depending on the availability of hosts in the studied area [24]. These variations suggest that environmental factors, local fauna composition, and even intraspecific aspects may influence both host preference and feeding behavior in sand flies.

The use of insecticide-impregnated collars has been widely recommended as a key strategy for reducing the transmission of VL in both humans and dogs, primarily by repelling sand fly vectors before they are able to feed [24,36,37]. Our study, conducted in Mariápolis, represents the first report on the feeding behavior of *Lu. longipalpis* in the State of São Paulo during the implementation of a deltamethrin-impregnated dog collar intervention. We confirmed the anthropophilic feeding tendency of this vector, with humans identified as the predominant source of blood. This finding is consistent with previous studies conducted in Pernambuco and Minas Gerais, where humans were also reported as the primary blood source for *Lu. longipalpis* females [38,39].

Chickens also emerged as an important blood source in our study, consistent with the findings from other authors [40,41], further highlighting their role in peridomestic environments where they may help sustain sand fly populations. In addition, we detected *Lu. longipalpis* females that had fed on dogs, the primary reservoir host in the urban transmission cycle of VL [42]. However, the relatively low proportion of *Lu. longipalpis* with dog-derived blood meals may suggest a potential impact of the dog collar intervention program, since 100% of the dogs present at the sand fly collection sites were collared.

Our study has some limitations that should be acknowledged. The first and most important is the lack of data for comparison about infection and sources of blood meal from a period before the dogs were collared. Secondly, it should be mentioned that local factors, such as the presence of other animals, can affect the feeding preferences of sand flies, making it difficult to compare findings across different studies and places.

Despite these limitations, our results are important. We found two *L. infantum*-positive sand flies that were collected from the same residence, albeit in different years (June 2023 and January 2024). At this address, six dogs were fitted with deltamethrin-impregnated collars, and none tested positive for VL during the study period. However, four dogs in the surroundings tested positive for LV by ELISA, two during the first collaring cycle (August 2022) and two during the third (August 2023), and may represent a probable source of infection. Interestingly, 81% of the engorged *Lu. longipalpis* females collected at this location had fed on chickens, while only 15% had fed on dogs. Although chickens are refractory to *Leishmania* infection [43], our findings confirm they are frequently a source of blood meal for *Lu. longipalpis*, underscoring their ecological importance in the feeding dynamics of this vector and raising important questions regarding the overall effectiveness of the canine collar program in interrupting transmission.

## 5. Conclusions

In conclusion, the study provides valuable insights into the feeding behavior and infection rates of sand flies in an endemic region. The high prevalence of mixed blood meals suggests complex transmission dynamics involving multiple hosts, emphasizing the importance of targeted vector control measures. The detection of *Leishmania* spp. DNA, even at a low prevalence, underscores the persistent risk of transmission, reinforcing the need for integrated approaches combining entomological monitoring, public health interventions, and community awareness to effectively manage leishmaniasis in affected areas.

## Figures and Tables

**Table 1:** Frequency of female sand fly specimens by capture method in Mariápolis, SP.

Capture Method	Number of Sand Flies *	Frequency (%)	95% CI **
Manual aspiration	378	91.3	88.06–93.76
CDC electric traps	32	7.73	5.43–10.85
Shannon trap	4	0.97	0.31–2.63
Total	414		

* χ^2^ = 628.93, df = 2, *p*-value < 2.2 × 10^−16^; ** 95% confidence interval for frequency parameters.

**Table 2:** Food sources identified in engorged *Lutzomyia longipalpis* females collected in Mariápolis between November 2022 and May 2024.

Blood Meal	Number of Sand Flies	(%)	Sources	Number of Sand Flies	(%)
Single			Human	48/113	42.5
	113	54.8	Chicken	50/113	44.2
			Dog	8/113	7.1
			Duck	7/113	6.2
Double	76		Human/chicken	62/76	81.6
		36.9	Chicken/dog	3/76	3.9
			Human/dog	11/76	14.5
Triple	17	8.3	Human/chicken/dog		
Total	206	100			

## Data Availability

The original contributions presented in this study are included in the article. Further inquiries can be directed to the corresponding author.
